# Effect of psychological intervention combined with feedback-based health education on lung cancer patients

**DOI:** 10.1590/1980-220X-REEUSP-2024-0170en

**Published:** 2025-03-03

**Authors:** Ying Huang, Xiaoxiao Xu, Feifei Weng, Chunxia Su

**Affiliations:** 1 Tongji University, Shanghai Pulmonary Hospital, Department of Oncology, Shanghai, China.; 2 Taizhou Hospital of Zhejiang Province affiliated to Wenzhou Medical University, Linhai, China.

**Keywords:** Psychological intervention, Teach-Back Communication, Lung Neoplasms, Risk Management, Intervenção Psicossocial, Comunicação para Apreensão de Informação, Neoplasias Pulmonares, Gestão de Riscos, Intervención Psicosocial, Método Teach-Back, Neoplasias Pulmonares, Gestión de Riesgos

## Abstract

**Objective:**

To explore the application effect of Psychological Intervention based on the emotional adaptation theory combined with Feedback-based Health Education (PICFHE) on lung cancer patients.

**Method:**

A total of 216 lung cancer patients were divided into a control group and an observation group using a random number table method. The control group received routine nursing intervention, while the observation group received PICFHE on the basis of the control group. Psychological status was compared using the Self Rating Anxiety Scale (SAS) and Self Rating Depression Scale (SDS). Quality of life was measured using WHOQOL-BREF scale and the respiratory condition, satisfaction with the nursing staff, and incidence of adverse reactions were observed. Data were analyzed quantitatively using the software SPSS 20.0.

**Results:**

After intervention, scores of SAS and SDS, respiratory rate, and total incidence of adverse reactions in the observation group were significantly lower than those in the control group. After intervention, the level of maximum volume ventilation and the scores in all dimensions of the WHOQOL-BREF scale of the observation group were higher than those of the control group.

**Conclusion:**

PICFHE can improve patients’ psychological status, their quality of life and satisfaction with the nursing staff, and reduce the incidence of adverse reactions.

## RESUMO

**Objetivo:**

Explorar o efeito da aplicação de Intervenção Psicológica baseada na teoria da adaptação emocional combinada com Educação para a Saúde baseada em Feedback (PICFHE) em pacientes com câncer de pulmão.

**Métodos:**

Um total de 216 pacientes com câncer de pulmão foi dividido em um grupo de controle e um grupo de observação usando um método de tabela de números aleatórios. O grupo controle recebeu intervenção de enfermagem de rotina, enquanto o grupo de observação recebeu PICFHE com base no grupo controle. O estado psicológico foi comparado usando a Escala de Autoavaliação de Ansiedade (SAS) e a Escala de Autoavaliação de Depressão (SDS), a qualidade de vida medida pela escala WHOQOL-BREF, e a condição respiratória, a satisfação com a enfermagem e a incidência de reações adversas foram observadas. Os dados foram analisados quantitativamente utilizando o software SPSS 20.0.

**Resultados:**

Após a intervenção, os escores de SAS e SDS, frequência respiratória e incidência total de reações adversas no grupo de observação foram significativamente menores do que no grupo de controle. Após a intervenção, o nível de ventilação de volume máximo e os escores em todas as dimensões da escala WHOQOL-BREF do grupo de observação foram maiores do que os do grupo controle.

**Conclusão:**

O PICFHE pode melhorar o estado psicológico, a qualidade de vida e a satisfação com a enfermagem, além de reduzir a incidência de reações adversas.

## RESUMEN

**Objetivo:**

Explorar el efecto de la aplicación de Intervención Psicológica basada en la teoría de la adaptación emocional combinada con educación para la salud basada en retroalimentación (PICFHE) en pacientes con cáncer de pulmón.

**Método:**

Un total de 216 pacientes con cáncer de pulmón se dividieron en un grupo de control y un grupo de observación utilizando un método de tabla de números aleatorios. El grupo de control recibió intervención de enfermería de rutina, mientras que el grupo de observación recibió PICFHE sobre la base del grupo de control. Se comparó el estado psicológico utilizando la Escala de autoevaluación de ansiedad (SAS) y la Escala de autoevaluación de depresión (SDS), se midió la calidad de vida utilizando la escala WHOQOL-BREF y se observaron la condición respiratoria, la satisfacción con el equipo de enfermería y la incidencia de reacciones adversas. Los datos fueron analizados cuantitativamente utilizando el software SPSS 20.0.

**Resultados:**

Después de la intervención, las puntuaciones de SAS y SDS, la frecuencia respiratoria y la incidencia total de reacciones adversas en el grupo de observación fueron significativamente inferiores a las del grupo de control. Después de la intervención, el nivel de ventilación de volumen máximo y las puntuaciones en todas las dimensiones de la escala WHOQOL-BREF del grupo de observación fueron superiores a los del grupo control.

**Conclusión:**

El PICFHE puede mejorar el estado psicológico, su calidad de vida y la satisfacción con el equipo de enfermería, y reducir la incidencia de reacciones adversas.

## INTRODUCTION

Lung cancer is the most common malignant tumor in clinical practice, with incidence rate and mortality ranking first, which seriously threatens human life and health^(1)^. At present, multiple methods can be used for the treatment of lung cancer, and clinical practice often adopts a multidisciplinary and comprehensive treatment model, emphasizing personalized treatment^(2,3)^. Comprehensive treatment methods such as surgery, chemotherapy, radiotherapy, and biological targeting have significantly improved clinical efficacy and patients’ quality of life^(4,5)^. However, lung cancer patients’ obvious concerns and fears about the disease can increase their physical and mental burden, easily generate negative emotions, affect their treatment compliance, and significantly affect their prognosis and quality of life^(6,7)^. Therefore, it is necessary to provide effective psychological intervention for lung cancer patients.

Conventional nursing is mainly focused on diseases, and health education is often orally provided^(8)^. The emphasis on psychological aspects is not high, and patients’ acceptance is low, resulting in limited intervention effectiveness^(9)^. The theory of emotional adaptation refers to the psychological process in which human emotional responses to repetitive or persistent stimuli gradually weaken. Psychological intervention based on the theory of emotional adaptation is a nursing intervention model aimed at the patients’ psychological changes at different periods^(10)^. Feedback-based health education is a new type of health education model that can deepen the impression of health knowledge and improve patients’ mastery of health education content through mutual feedback between nursing staff and patients^(11,12)^. Therefore, we propose the hypothesis that application effect of Psychological Intervention based on emotional adaptation theory combined with Feedback-based Health Education (PICFHE) on lung cancer patients can bring them more benefits. We hope that this model can better help improve the quality of life of lung cancer patients. We demonstrate the effectiveness of our psychological model for lung cancer patients by comparing their psychological status, respiratory condition, quality of life, nursing satisfaction, and incidence of adverse reactions. Thus, the present study aimed to explore the effect of applying Psychological Intervention based on the theory of emotional adaptation combined with Feedback-Based Health Education (PICFHE) in patients with lung cancer.

## METHOD

In this study, to explore the effectiveness of PICFHE on lung cancer patients compared to conventional nursing, we divided 216 lung cancer patients into two groups. The control group received routine nursing intervention, while the observation group received PICFHE on the basis of the control group. Next, we compared the psychological status, respiratory condition, quality of life, nursing satisfaction, and incidence of adverse reactions between the two groups of patients. The specific content is reported as follows.

### Study Design

This study selected a small sample size of lung cancer patients and is a pilot study, and it is a comparative study of a new psychological care model (PICFHE) for lung cancer patients in our hospital. The control group received routine nursing intervention. Nursing staff provides oral health education to patients, as well as corresponding prevention and treatment measures for radiation and chemotherapy, and guidance on diet and medication. During this period, psychological counseling is provided and the intervention is given for 3 months.

The observation group implemented psychological intervention based on the emotional adaptation theory, combined with feedback-based health education on the basis of the control group, with an intervention period of 3 months. The specific content is as follows:

(1) Psychological intervention based on emotional adaptation theory^(13,14,15)^. ① Denial stage: In the early stage of diagnosis of lung cancer, the patient’s psychological status is in the denial stage. Through videos, pictures, and other means, patients can increase their understanding of the disease, being informed that lung cancer is not an incurable disease, and timely treatment is the key to save lives. ② In the early and middle stages of lung cancer, the progression of the disease can be controlled through surgery or radiotherapy and chemotherapy. Anger stage: After lung cancer surgery or during radiotherapy and chemotherapy, patients are prone to develop angry emotions due to toxic side effects or surgical trauma stimuli. The nursing staff has to help patients choose appropriate ways of venting based on their psychological characteristics, such as confiding in family or friends, writing a diary, etc. ③ Compromise stage: After surgery and during the initial stage of radiotherapy and chemotherapy, patients have the strongest desire for survival. Nursing staff has to convey disease knowledge and relevant precautions to patients during this stage to improve their health beliefs; at the same time, we will focus on teaching patients practical skills related to self-care, such as diet matching, techniques for preventing and alleviating toxic and side effects, and medication knowledge, to improve their healthy behavior. Despair stage: After a long period of radiotherapy and chemotherapy, if the condition remains uncontrollable or serious side effects occur, patients may develop a sense of despair and even give up treatment. At this time, nursing staff can be encouraged to communicate more with their patients to enhance their treatment confidence by citing successful cases of previous treatment, similar situations, and ultimately successful cases of treatment. ④ Acceptance stage: At this stage, patients have a high level of cooperation and need to be encouraged and praised. Music therapy, meditation training, and other methods can be used to help them maintain emotional relaxation.

(2) Feedback-based health education^(16,17,18)^. ① Assessment: Collect the basic information of patients, understand their disease cognition, psychological status, personality and education level through Structured interview, and formulate intervention plans according to the interview results. ② Health education: Carry out corresponding health education according to the intervention plan, mainly through video playback and PPT to introduce patients to the occurrence and development of lung cancer, the advantages and disadvantages of radical resection surgery, radiation and chemotherapy knowledge, side reaction prevention, relevant precautions, etc., emphasizing the relationship between psychology and disease rehabilitation, and demonstrating the skills and steps of side reaction prevention and treatment to patients. After the health education content is completed, patients are required to retell or demonstrate the relevant content, and based on the feedback results, repeat the preaching or demonstration until they fully grasp it. ③ Positive feedback: Patients are required to keep a daily diary, record meaningful events that occurred on that day, participate in a weekly patient gathering, and provide feedback on their gains and experiences during the activity. Nursing staff patiently answers patients’ feedback every week, inquires about health knowledge and behavioral skills that patients are required to master during daily rounds, and conducts a weekly reassessment and re-education based on the feedback, with a cycle of follow-up. After discharge, patients can receive evaluation and feedback-based health education through phone and WeChat.

### Sample Definition

A total of 216 lung cancer patients admitted to Shanghai Pulmonary Hospital from February 2022 to January 2023 were selected. All the lung cancer patients were divided into a control group and an observation group using a random number table method, with 108 patients in each group. The control group received routine nursing intervention, while the observation group received psychological intervention based on emotional adaptation theory combined with feedback-based health education on the basis of the control group.

### Selection Criteria

Inclusion criteria: ① Meeting the diagnostic criteria for lung cancer and receiving chemotherapy; ② Stable condition, certain cognitive abilities, and good communication skills; ③ Good compliance. Exclusion criteria: ① Critical condition with unstable vital signs; ② Concomitant with other tumors or serious internal medical diseases; ③ Having cognitive and mental impairments, unable to communicate normally.

### Outcomes and Data Collection Instruments

#### Observation Indicators and Evaluation Standards

① Before and after intervention, the psychological status of two groups of patients was evaluated using the Self Rating Anxiety Scale (SAS)^(19,20)^ and Self Rating Depression Scale (SDS)^(21,22)^, both containing 20 items. A 4-level scoring system was used, with higher scores indicating poorer psychological status.

② Before and after intervention, compare the respiratory status of two groups of patients, including respiratory rate, venous oxygen saturation, and maximum volume ventilation (MVV). ③ Before and after intervention, the World Health Organization Quality of Life Instrument, short form (WHO QOL BREF)^(23)^, was used to evaluate the quality of life of the two groups of patients, including 26 items in four dimensions: physiological function, psychological function, social function, and environmental function. Each item was scored 1–5 points, and the higher the score, the better the quality of life. ④ After intervention, the satisfaction with the nursing staff of the two groups of patients was evaluated using a hospital made satisfaction survey scale. ⑤ Compare the incidence of adverse reactions between the two groups of patients, including loss of appetite, nausea and vomiting, and abdominal distension.

Nursing satisfaction scale.^(24,25)^

The scale includes the 10 items below, rated 0–10 points, with higher scores indicating higher satisfaction with the nursing staff. It was subjected to a pilot test and daily use in our hospital.

1. Is there a nurse responsible for taking care of you during your hospitalization?
2. Did the ward nurse introduce you to the environmental facilities in the ward when you were admitted?
3. Does the nurse proactively verify your name, bed number, and other relevant information before administering medication/performing procedures on you?
4. Can nurses comfort and help you when you are sad or anxious?
5. Can nurses understand your condition while taking care of you, making you as comfortable and painless as possible?
6. Can nurses also promptly monitor the ward and address your needs when you are not using the red light call?
7. Is the nurse skilled in performing the procedures for you?
8. Before performing nursing procedures, can the nurse inform you of the purpose and precautions of this operation?
9. During nursing treatment, do nurses use curtains, screens, and other shielding methods to protect your privacy?
10. When your illness makes you unable to take care of yourself, will the nurse provide timely life care for you?

### Data Analysis

The software SPSS 20.0 was used for statistical analysis of all data, with counting data expressed in terms of number of cases and rate (%). Inter group comparisons were conducted using χ^2^ Inspection; Measurement data were presented as mean ± standard deviation $\left(\bar{\text{x}}\pm \text{s}\right)$ , and t-test is used for inter group comparison; The difference was statistically significant with P < 0.05.

### Ethical Aspects

This study was approved by the hospital ethics committee (K22–013Y), and all patients signed an informed consent form.

## RESULTS

### General Information of The Patient

A total of 216 lung cancer patients were divided into two groups randomly. In the control group, there were 65 men and 43 women; age ranged from 44 to 79 years, with an average age of (58.64 ± 6.69) years; the pathological types were: 5 cases of small-cell carcinoma, 60 cases of squamous cell carcinoma, 43 cases of adenocarcinoma. Clinical staging was characterized by 22 cases in stage II, 50 cases in stage III, and 36 cases in stage IV. Education level ranged from junior high school and below (40 cases) to high school and above (68 cases). In the observation group, there were 63 men and 45 women; age ranged from 43 to 78 years, with an average age of (59.14 ± 7.83) years; the pathological types were 6 cases of small-cell carcinoma, 61 cases of squamous cell carcinoma, 41 cases of adenocarcinoma. Clinical staging had 21 cases in stage II, 52 cases in stage III, and 35 cases in stage IV. Education level had 39 cases of junior high school and below, 69 cases of high school and above. There was no statistically significant difference in baseline characteristics such as gender, age, and clinical stage between the two groups of patients (P > 0.05), indicating comparability.

### Comparison of Psychological Status

Before intervention, there was no statistically significant difference in SAS and SDS scores between the two groups of patients (P > 0.05) (Table 1). After the intervention, the SAS and SDS scores of the two groups of patients were significantly lower than before the intervention (Table 1), with those of the observation group being significantly lower than those of the control group, with statistical significance (P < 0.01).

**Table 1:** Comparison of SAS and SDS scales scores between two groups of patients before and after intervention – Shanghai, China, 2022/2023.

Scale	Time	Observation arm $\left(\text{n}\;\text{=}\;\text{108,}\;\bar{\text{x}}\;\pm \text{s}\right)$	Control arm $\left(\text{n}\;\text{=}\;\text{108,}\;\bar{\text{x}}\;\pm \text{s}\right)$	t-value	p-value
SAS	Before intervention	62.87 ± 9.67	62.48 ± 10.21	0.286	0.775
	After intervention	44.18 ± 5.40*	50.24 ± 6.37*	−7.509	0.000
SDS	Before intervention	61.32 ± 10.52	62.43 ± 7.00	−0.902	0.368
	After intervention	43.42 ± 6.36*	49.32 ± 8.16*	−5.904	0.000

Note: *Compared with the condition before the intervention in this group, P < 0.01.

### Comparison of Respiratory Conditions

Before the intervention, there was no statistically significant difference in respiratory rate, venous oxygen saturation, and MVV between the two groups of patients (P > 0.05). After the intervention, the respiratory frequency of both groups of patients was lower than before, and the MVV was higher than before the intervention (Table 2). The venous oxygen saturation of the observation group was higher than before the intervention, and the differences were statistically significant (P < 0.05). After the intervention, the respiratory rate of the observation group was lower than that of the control group, while the MVV level was higher, with statistical significance (P < 0.05) (Table 2). However, there was no statistically significant difference in venous oxygen saturation between the two groups of patients (P > 0.05).

**Table 2:** Comparison of respiratory status between two groups of patients before and after intervention – Shanghai, China, 2022/2023.

Parameter	Time	Observation arm $\left(\text{n}\;\text{=}\;\text{105,}\;\bar{\text{x}}\;\pm \text{s}\right)$	Control arm $\left(\text{n}\;\text{=}\;\text{108,}\;\bar{\text{x}}\;\pm \text{s}\right)$	t-value	p-value
Respiratory rate (Times/minute)	Before intervention	20.31 ± 2.72	20.26 ± 2.87	0.145	0.885
	After intervention	16.98 ± 2.25*	19.11 ± 3.06	−5.803	0.000
Venous oxygen saturation (%)	Before intervention	91.31 ± 4.31	90.74 ± 5.13	0.886	0.376
	After intervention	99.34 ± 5.68*	95.89 ± 4.07	5.110	0.000
MVV(L)	Before intervention	52.41 ± 8.59	53.38 ± 8.64	−0.820	0.413
	After intervention	64.38 ± 9.27*	59.38 ± 8.97*	4.015	0.000

Note: *Compared with before intervention in this group, P < 0.05.

### Comparison of Quality of Life

Before intervention, there was no statistically significant difference in the scores and total scores of the WHOQOL-BREF scale between the two groups of patients (P > 0.05) (Table 3). After the intervention, the scores and total scores of each dimension of the WHOQOL-BREF scale in both groups of patients were higher than before the intervention, and also higher in the observation group compared to the control group, with statistical significance (P < 0.05).

**Table 3:** Comparison of WHOQOL-BREF scale scores between two groups of patients before and after intervention – Shanghai, China, 2022/2023.

Dimension	Time	Observation arm $\left(\text{n}\;\text{=}\;\text{108,}\;\bar{\text{x}}\;\pm \text{s}\right)$	Control arm $\left(\text{n}\;\text{=}\;\text{108,}\;\bar{\text{x}}\;\pm \text{s}\right)$	t-value	p-value
Physiologic function	Before intervention	21.47 ± 2.38	22.00 ± 2.05	−1.739	0.084
	After intervention	26.39 ± 2.15*	23.67 ± 2.91	7.778	0.000
Psychological function	Before intervention	19.68 ± 3.20	19.39 ± 2.02	0.785	0.433
	After intervention	26.31 ± 2.23*	22.99 ± 3.09*	8.988	0.000
Social function	Before intervention	8.34 ± 1.69	8.28 ± 1.28	0.316	0.752
	After intervention	12.31 ± 1.37*	10.35 ± 1.28*	10.084	0.000
Environmental function	Before intervention	25.32 ± 2.57	25.53 ± 3.00	−0.533	0.594
	After intervention	33.31 ± 3.00*	30.16 ± 3.22*	7.398	0.000
Total score	Before intervention	74.81 ± 6.30	73.19 ± 4.98	−0.489	0.625
	After intervention	98.31 ± 5.72*	87.17 ± 7.37*	12.357	0.000

Note: *Compared with before intervention in this group, P < 0.05.

### Comparison of Nursing Satisfaction

After intervention, the score of satisfaction with the nursing staff of the observation group patients was (96.53 ± 15.13) points, which was higher than the control group patients’ (88.15 ± 12.25) points, and the difference was statistically significant (*t* = 4.451, *P* = 0.000).

### Comparison of Adverse Reactions

After intervention, 7 cases of anorexia, 11 cases of nausea and vomiting, and 5 cases of abdominal distension occurred in the observation group, with a total incidence of adverse reactions of 21.30% (23/108); the control group experienced 14 cases of anorexia, 25 cases of nausea and vomiting, and 8 cases of abdominal distension, with a total incidence of adverse reactions of 43.52% (47/108); the total incidence of adverse reactions in the observation group was lower than that in the control group, and the difference was statistically significant (χ^2^ = 4.089, P = 0.039).

## DISCUSSION

In the study, after PICFHE, the SAS and SDS scores in the observation group were significantly lower than those in the control group, the respiratory rate of the observation group patients was lower and the MVV level was higher. The scores and total scores of each dimension of the WHOQOL-BREF in the observation group were higher than those in the control group. After PICFHE, the score of satisfaction with the nursing staff of the observation group was higher compared to the control group, while the overall incidence of adverse reactions was lower. Therefore, PICFHE has improved the psychological and respiratory status of lung cancer patients, enhanced their quality of life and satisfaction with the nursing staff, and reduced the incidence of adverse reactions.

Lung cancer is a malignant tumor that progresses rapidly and has a high 5-year mortality rate^(26)^. At present, clinical treatment for early and middle stage lung cancer mainly adopts surgical resection and postoperative radiotherapy and chemotherapy^(27)^. Although the treatment can effectively control the progression of the disease and prolong the patient’s survival time, during its process, due to patients’ limited understanding of the disease, as well as the impact of surgical trauma, radiotherapy and chemotherapy toxic side effects, they inevitably develop negative psychological aspects, which lead to a decrease in treatment compliance, affecting quality of life and hindering the development of treatment and nursing work^(28,29)^. Conventional nursing interventions mainly focus on physiological diseases, lacking targeted interventions^(30)^. In addition, most lung cancer patients are middle-aged and older, with poor information reception ability, which makes it difficult to achieve the expected results^(31)^. Therefore, it can be seen that scientific and effective nursing interventions need to be implemented based on the characteristics of lung cancer patients to enhance their treatment confidence and improve their prognosis.

The theory of emotional adaptation believes that when humans experience certain strong stimuli, the strong stimuli gradually fade over time, and such changes are called emotional adaptation^(32)^; feedback-based education is formed under this theory, through the process of health education and retelling, to achieve two-way effective transmission of information between nurses and patients, continuously increasing patients’ understanding of the disease and improving their mastery level(33).

The results of this study showed that after the intervention, the SAS and SDS scores of both groups of patients were significantly lower than before the intervention, and the SAS and SDS scores of the observation group were significantly lower than those of the control group, indicating that psychological intervention based on the emotional adaptation theory combined with feedback-based health education can improve the psychological status of lung cancer patients. This is because during the intervention process, nursing staff always motivate patients through words, behaviors, and other means, acknowledging their efforts and expressing sincere appreciation for their progress.

The results of this study show that psychological intervention based on emotional adaptation theory combined with feedback-based health education can improve the respiratory status of lung cancer patients. This is because through systematic health education, patients can understand the importance of respiratory training and master its correct methods. During respiratory training, patients are encouraged to recognize every move, appreciate the completion of their goals, and actively complete the exercise^(34,35)^. Patients who spontaneously participate in respiratory training and engage in moderate aerobic exercise can effectively improve their cardiopulmonary function, thereby improving their respiratory condition^(34,35)^.

In recent years, improving the quality of life of patients with malignant tumors has gradually attracted attention, and quality of life has become an important indicator for evaluating clinical efficacy^(36,37)^. Previous studies have shown that taking intervention measures to improve patients’ cognitive level of the disease, reduce negative emotions, and improve compliance behavior can all improve the quality of life of lung cancer patients^(36,37)^. The results of this study show that psychological intervention based on emotional adaptation theory, combined with feedback-based health education, can improve the quality of life of lung cancer patients. This is because this combination can significantly improve patients’ disease cognition level, provide patients with positive psychological intervention, and enable them to maintain a good mentality, actively engage in rehabilitation exercises, which are conducive to improving patients’ physical functions, thereby improving their quality of life.

The results of this study also show that the intervention above-mentioned can improve the nursing satisfaction of lung cancer patients. This is because throughout the nursing process, it is necessary to dynamically evaluate the patient’s status, timely identify their health problems, actively encourage them during communication, patiently answer questions and clarify doubts, correct their bad behavior, acknowledge their slight progress, and improve their level of hope, stimulating patients’ enthusiasm, enabling them to independently learn about health, establish a healthy concept, alleviate negative emotions, improve compliance behavior, and always focus on patients when communicating. Caring for patients can establish a good nurse-patient relationship, thereby improving the nursing satisfaction of lung cancer patients.

The advancements of this study are that psychological intervention based on emotional adaptation theory, combined with feedback-based health education, alleviated patients’ negative emotions, kept them happy, improved their health awareness, treatment compliance, and treatment tolerance, and reduced the risk of adverse reactions. The drawback of this study is that the sample size is not large enough, and the cases used were only from a hospital in Shanghai, China, which belongs to a single center study. In addition, receiving different treatment plans for two groups of lung cancer patients may also affect their psychological well-being, thereby affecting the results of this study. We also did not consider the difference in families’ economic situations among the two groups of patients.

## CONCLUSION

In summary, psychological intervention based on emotional adaptation theory, combined with feedback-based health education, can alleviate the negative emotions of lung cancer patients, improve their psychological and respiratory conditions, encourage them to actively participate in rehabilitation exercises, improve their compliance behavior, reduce the incidence of adverse reactions, and improve their quality of life and satisfaction with the nursing staff.
